# Toads and Warts

**Published:** 1913-06

**Authors:** 


### Toads and Warts.

We have all heard the old grannies say that warts were due to handling toads, and have laughed at the notion. Quite a long time ago we disproved this statement by personal experiment, but we also proved that rhus toxicodendron was harmless—to the same subject of experiment. In the light of recent discoveries showing the presence of an irritant poison—buffonin—in the skin of the toad, and a similar one in the frog, we are rather inclined to place some credence in the toad theory of the production of warts. Caspar and Loewy have reported that the arrow poison of the Indians of Columbia is derived by pricking the skin of a frog. This poisoning paralyzes the animal shot and enables him to be captured even if slightly wounded. Now don't understand us to claim that all warts are due to handling toads, or frogs, nor that every child handling a bactrian develops warts. But papillomas generally are due to irritation, perhaps especially of a chemic nature. Warts are undeniably more common in country dwellers than in city dwellers, they are more common in the summer than in the winter, though often persistent, they are more common in the young and rarely affect adults who are careful as to their hands. We suggest that, this summer, our readers investigate the etiology clinically, with a view to determining how far exposure to various irritants is operative, and with particular attention to the toad theory and to ascertain other potential causes.

